# 5,7-Dimethyl-2,3-dihydro-1*H*-1,4-diazepin-4-ium picrate

**DOI:** 10.1107/S160053681001487X

**Published:** 2010-04-28

**Authors:** Jerry P. Jasinski, Ray J. Butcher, H. S. Yathirajan, B. Narayana, K. Prakash Kamath

**Affiliations:** a Department of Chemistry, Keene State College, 229 Main Street, Keene, NH 03435-2001, USA; b Department of Chemistry, Howard University, 525 College Street NW, Washington, DC 20059, USA; c Department of Studies in Chemistry, University of Mysore, Manasagangotri, Mysore 570 006, India; d Department of Studies in Chemistry, Mangalore University, Mangalagangotri, 574 199, India

## Abstract

In the cation of the title compound, C_7_H_13_N_2_^+^·C_6_H_2_N_3_O_7_^−^, the seven-membered 1,4-diazepine ring forms a twist chair conformation. The two *o*-nitro groups in the anion are twisted by 35.0 (7) and 36.0 (9)° from the benzene ring. In the crystal, N—H⋯O hydrogen bonds between the cation and anion along with weak C—H⋯O hydrogen bonds produce chains along the *b* axis. C—H⋯O hydrogen bonds connecting the chains are also present.

## Related literature

For biological applications of 1,4-diazepine derivatives, see: Andrews *et al.* (2001 ▶); Block *et al.* (1989 ▶); Carp (1999 ▶); Moroz (2004 ▶). For treatment of CNS disorders, see: Walser *et al.* (1978 ▶). For pharmacological profiles, see: Carlos *et al.* (2004 ▶). For related structures, see: Ferguson *et al.* (1990 ▶); Harrison *et al.* (2005 ▶); Peeters *et al.* (1997 ▶); Petcher *et al.* (1985 ▶); Rashid *et al.* (2006 ▶); Yang *et al.* (2007 ▶). For density functional theory calculations, see: Schmidt & Polik (2007 ▶); Hehre *et al.* (1986 ▶).
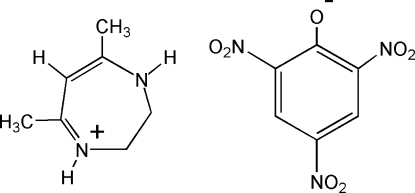


## Experimental

### 

#### Crystal data

C_7_H_13_N_2_^+^·C_6_H_2_N_3_O^7−^
*M* *_r_* = 353.30
Monoclinic, 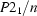

*a* = 7.2341 (3) Å
*b* = 27.6458 (6) Å
*c* = 8.2831 (3) Å
β = 110.611 (4)°
*V* = 1550.52 (9) Å^3^
*Z* = 4
Mo *K*α radiation
μ = 0.13 mm^−1^
*T* = 200 K
0.45 × 0.37 × 0.24 mm

#### Data collection

Oxford Diffraction Gemini diffractometer
Absorption correction: multi-scan (*CrysAlis RED*; Oxford Diffraction, 2007 ▶) *T*_min_ = 0.962, *T*_max_ = 0.970
24773 measured reflections
6353 independent reflections
4493 reflections with *I* > 2σ(*I*)
*R*_int_ = 0.028

#### Refinement

*R*[*F* ^2^ > 2σ(*F* ^2^)] = 0.056
*wR*(*F* ^2^) = 0.150
*S* = 1.04
6353 reflections
228 parameters
H-atom parameters constrained
Δρ_max_ = 0.41 e Å^−3^
Δρ_min_ = −0.23 e Å^−3^

### 

Data collection: *CrysAlis PRO* (Oxford Diffraction, 2007 ▶); cell refinement: *CrysAlis RED* (Oxford Diffraction, 2007 ▶); data reduction: *CrysAlis RED*; program(s) used to solve structure: *SHELXS97* (Sheldrick, 2008 ▶); program(s) used to refine structure: *SHELXL97* (Sheldrick, 2008 ▶); molecular graphics: *SHELXTL* (Sheldrick, 2008 ▶); software used to prepare material for publication: *SHELXTL* and *PLATON* (Spek, 2009 ▶).

## Supplementary Material

Crystal structure: contains datablocks global, I. DOI: 10.1107/S160053681001487X/is2536sup1.cif

Structure factors: contains datablocks I. DOI: 10.1107/S160053681001487X/is2536Isup2.hkl

Additional supplementary materials:  crystallographic information; 3D view; checkCIF report

## Figures and Tables

**Table 1:** Hydrogen-bond geometry (Å, °)

*D*—H⋯*A*	*D*—H	H⋯*A*	*D*⋯*A*	*D*—H⋯*A*
N1*B*—H1*BC*⋯O1*A*	0.88	1.98	2.8434 (13)	169
N2*B*—H2*BC*⋯O42*A*^i^	0.88	2.09	2.9657 (14)	176
C5*A*—H5*AA*⋯O22*A*^ii^	0.95	2.54	3.4570 (16)	162
C7*B*—H7*BA*⋯O1*A*	0.98	2.55	3.2817 (17)	131
C1*B*—H1*BA*⋯O62*A*	0.99	2.45	3.2861 (15)	142
C1*B*—H1*BB*⋯O1*A*^iii^	0.99	2.51	3.4477 (16)	158
C2*B*—H2*BA*⋯O61*A*^iv^	0.99	2.48	3.0678 (16)	117
C2*B*—H2*BB*⋯O1*A*^v^	0.99	2.46	3.3135 (16)	144
C4*B*—H4*BA*⋯O42*A*^i^	0.98	2.59	3.4692 (17)	149

Symmetry codes: (i) 
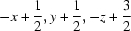
; (ii) 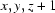; (iii) 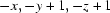; (iv) 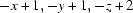; (v) 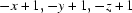.

**Fig. 1.**
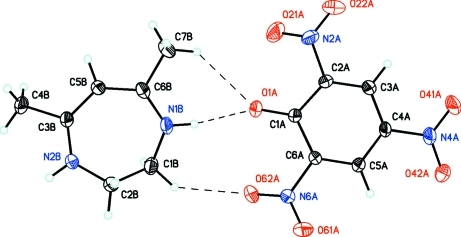
Molecular structure of the C7H13N2+.C6H2N3O7- cation-anion pair, showing the atom labeling scheme and 50% probability displacement ellipsoids. Dashed lines indicate intermolecular N—H···O hydrogen bonds and weak C—H···O hydrogen bond interactions.

**Fig. 2.**
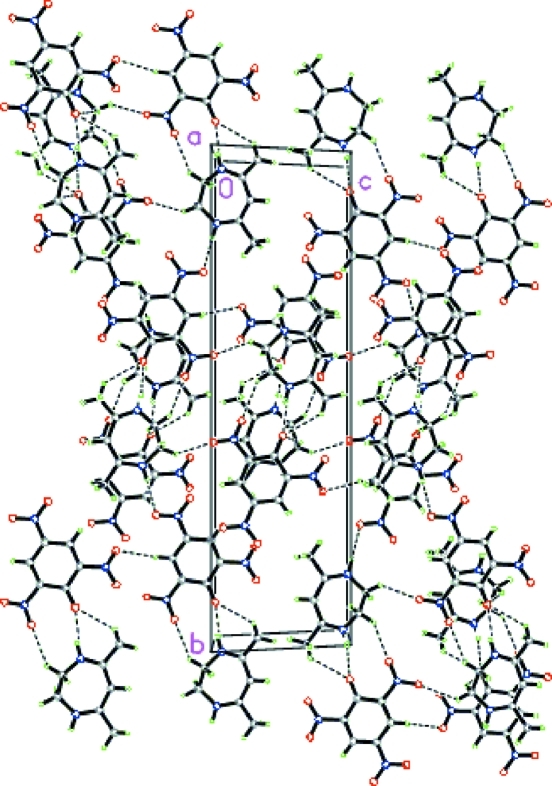
Packing diagram of the title compound, viewed down the a axis. Dashed lines indicate intermolecular N—H···O hydrogen bonds and weak C—H···O hydrogen bonds.

## supplementary crystallographic information

### Comment

1,4-Diazepine derivatives display tranquilizing, muscle-relaxant, anti-convulsant and sedative effects (Block *et al.*, 1989). Today many diazepine derivatives are widely used as daytime sedatives, tranquilizers, sleep inducers, anesthetics, anticonvulsants and muscle relaxants (Moroz, 2004). The use of this class of compounds with therapeutic purposes is not only confined to anxiety and stress conditions, given that minor changes in their structures can produce a host of different biological activities, and novel applications are continuously emerging (Andrews *et al.*, 2001; Carp, 1999). Five-atom heterocyclic fused benzodiazepine ring systems occupy a prominent place among drugs for treatment of CNS disorders (Walser *et al.*, 1978). The introduction of alprazolam, triazolam and midazolam in chemotherapy has enhanced the interest in the preparation of novel five-atom heterocyclic fused benzodiazepine ring systems. Numerous analogs of alprazolam, triazolam and midazolam have been described, and they have shown different pharmacological profiles related to those of their parent compounds (Carlos *et al.*, 2004). In continuation of our work on picrates of biologically important molecules, we have prepared a new picrate of 5,7-dimethyl-2,3-dihydro-1*H*-1,4-diazepine, C_7_H_13_N_2_^+^.C_6_H_2_N_3_O_7_^-^, and its crystal structure is reported.

The title compound, C_13_H_15_N_5_O_7_, crystallizes as a salt with one C_7_H_13_N_2_^+^.C_6_H_2_N_3_O_7_^-^ cation-anion pair in the asymmetric unit (Fig. 1). The dihedral angle between the mean planes of the benzene and 1,4-diazepine rings is 4.4 (6)°. In the cation, the seven membered 1,4-diazepine ring forms a twist chair conformation with Cs asymmetry parameters of -0.4004 and 0.3553°, for the sp^3^ hybridized C1B and C2B atoms, respectively. The two *o*-nitro groups in the anion are twisted by 35.0 (7) and 36.0 (9)° from the mean plane of the benzene ring. Bond distances and angles in both the cation and anion are in normal ranges. Cation-anion N—H···O hydrogen bonds [N1B—H1BC···O1A & N2B—H2BC···O42A] along with weak C—H···O intermolecular interactions (Table 1) produce a network of infinite N1B—H1BC···O1A/N2B—H2BC···O42A chains along the *b* axis which helps to establish crystal packing (Fig. 2).

A density functional theory (DFT) geometry optimization molecular orbital calculation (Schmidt & Polik, 2007) was performed on the independent cation-anion pair (C_7_H_13_N_2_^+^.C_6_H_2_N_3_O_7_^-^) within the asymmetric unit with the B3LYP/6-311+G(d,p) basis set (Hehre *et al.*, 1986). Starting geometries were taken from X-ray refinement data. The dihedral angle between the mean planes of the benzene and 1,4-diazepine rings increases to 30.9 (5)°. In the anion, the mean planes of the two *o*-nitro groups each become twisted by 35.5 (3)°, from the mean plane of the benzene ring. The mean plane of the *p*-nitro group remains planar to the benzene ring. These observations suggest that the N—H···O hydrogen bonds and weak C—H···O intermolecular interactions play a significant role in crystal stability.

### Experimental

5,7-Dimethyl-2,3-dihydro-1*H*-1,4-diazepine (1.24 g, 0.01 mol) was dissolved in 20 ml of alcohol. Picric acid (2.29 g, 0.01 mol) was dissolved in 40 ml of water. Both the solutions were mixed and to this, 5 ml of 3M HCl was added and stirred for few minutes. The formed complex was filtered and dried (m.p. 425 K). Composition: Found (calculated): C 44.15 (44.20), H 4.22 (4.28), N 19.78% (19.82%).

### Refinement

All of the H atoms were placed in their calculated positions and then refined using the riding model, with C—H = 0.95–0.99 Å and N—H = 0.88 Å, and with *U*_iso_(H) = 1.18–1.51*U*_eq_(C, N).

### Crystal data

**Table:**

C_7_H_13_N_2_^+^·C_6_H_2_N_3_O^7^^−^	*F*(000) = 736
*M**_r_* = 353.30	*D*_x_ = 1.513 Mg m^−^^3^
Monoclinic, *P*2_1_/*n*	Mo *K*α radiation, λ = 0.71073 Å
Hall symbol: -P 2yn	Cell parameters from 9544 reflections
*a* = 7.2341 (3) Å	θ = 4.7–34.7°
*b* = 27.6458 (6) Å	µ = 0.13 mm^−^^1^
*c* = 8.2831 (3) Å	*T* = 200 K
β = 110.611 (4)°	Chunk, colorless
*V* = 1550.52 (9) Å^3^	0.45 × 0.37 × 0.24 mm
*Z* = 4	

### Data collection

**Table:**

Oxford Diffraction Gemini diffractometer	6353 independent reflections
Radiation source: fine-focus sealed tube	4493 reflections with *I* > 2σ(*I*)
graphite	*R*_int_ = 0.028
Detector resolution: 10.5081 pixels mm^-1^	θ_max_ = 34.8°, θ_min_ = 4.7°
φ and ω scans	*h* = −11→11
Absorption correction: multi-scan (*CrysAlis RED*; Oxford Diffraction, 2007)	*k* = −43→44
*T*_min_ = 0.962, *T*_max_ = 0.970	*l* = −13→13
24773 measured reflections	

### Refinement

**Table:**

Refinement on *F*^2^	Primary atom site location: structure-invariant direct methods
Least-squares matrix: full	Secondary atom site location: difference Fourier map
*R*[*F*^2^ > 2σ(*F*^2^)] = 0.056	Hydrogen site location: inferred from neighbouring sites
*wR*(*F*^2^) = 0.150	H-atom parameters constrained
*S* = 1.04	*w* = 1/[σ^2^(*F*_o_^2^) + (0.074*P*)^2^ + 0.3582*P*] where *P* = (*F*_o_^2^ + 2*F*_c_^2^)/3
6353 reflections	(Δ/σ)_max_ = 0.001
228 parameters	Δρ_max_ = 0.41 e Å^−^^3^
0 restraints	Δρ_min_ = −0.23 e Å^−^^3^

### Special details

**Table:**

Geometry. All esds (except the esd in the dihedral angle between two l.s. planes) are estimated using the full covariance matrix. The cell esds are taken into account individually in the estimation of esds in distances, angles and torsion angles; correlations between esds in cell parameters are only used when they are defined by crystal symmetry. An approximate (isotropic) treatment of cell esds is used for estimating esds involving l.s. planes.
Refinement. Refinement of *F*^2^ against ALL reflections. The weighted *R*-factor *wR* and goodness of fit *S* are based on *F*^2^, conventional *R*-factors *R* are based on *F*, with *F* set to zero for negative *F*^2^. The threshold expression of *F*^2^ > σ(*F*^2^) is used only for calculating *R*-factors(gt) *etc*. and is not relevant to the choice of reflections for refinement. *R*-factors based on *F*^2^ are statistically about twice as large as those based on *F*, and *R*- factors based on ALL data will be even larger.

### Fractional atomic coordinates and isotropic or equivalent isotropic displacement parameters (Å^2^)

**Table:**

	*x*	*y*	*z*	*U*_iso_*/*U*_eq_	
O1A	0.30761 (14)	0.42368 (3)	0.49438 (11)	0.02584 (19)	
O21A	0.1166 (2)	0.37603 (5)	0.19050 (14)	0.0569 (4)	
O22A	0.2759 (3)	0.30970 (5)	0.20409 (16)	0.0696 (5)	
O41A	0.2539 (2)	0.20668 (4)	0.67603 (15)	0.0493 (3)	
O42A	0.2899 (2)	0.23953 (4)	0.92133 (14)	0.0444 (3)	
O61A	0.4592 (2)	0.41131 (4)	0.99837 (13)	0.0466 (3)	
O62A	0.26070 (17)	0.45405 (3)	0.79164 (13)	0.0374 (2)	
N2A	0.2123 (2)	0.34203 (4)	0.27069 (14)	0.0367 (3)	
N4A	0.27398 (18)	0.24249 (4)	0.76838 (15)	0.0295 (2)	
N6A	0.34729 (18)	0.41624 (4)	0.84935 (13)	0.0257 (2)	
C1A	0.28914 (16)	0.38355 (4)	0.55484 (14)	0.0188 (2)	
C2A	0.25139 (19)	0.33908 (4)	0.45574 (14)	0.0230 (2)	
C3A	0.2521 (2)	0.29387 (4)	0.52339 (15)	0.0247 (2)	
H3AA	0.2331	0.2660	0.4524	0.030*	
C4A	0.28112 (19)	0.28952 (4)	0.69802 (15)	0.0224 (2)	
C5A	0.31328 (18)	0.32993 (4)	0.80487 (15)	0.0213 (2)	
H5AA	0.3340	0.3265	0.9241	0.026*	
C6A	0.31432 (17)	0.37484 (4)	0.73403 (14)	0.0194 (2)	
C7B	0.2662 (3)	0.50023 (5)	0.18073 (19)	0.0357 (3)	
H7BA	0.2199	0.4691	0.2088	0.054*	
H7BB	0.4074	0.4981	0.1993	0.054*	
H7BC	0.1929	0.5082	0.0597	0.054*	
N1B	0.23246 (16)	0.52460 (3)	0.44657 (14)	0.0252 (2)	
H1BC	0.2648	0.4943	0.4762	0.030*	
N2B	0.24482 (17)	0.63760 (3)	0.48145 (13)	0.0250 (2)	
H2BC	0.2328	0.6674	0.5141	0.030*	
C1B	0.1824 (2)	0.55501 (4)	0.56818 (17)	0.0277 (3)	
H1BA	0.2014	0.5362	0.6746	0.033*	
H1BB	0.0409	0.5638	0.5178	0.033*	
C2B	0.3044 (2)	0.60105 (4)	0.61571 (16)	0.0267 (2)	
H2BA	0.2927	0.6146	0.7223	0.032*	
H2BB	0.4450	0.5929	0.6406	0.032*	
C3B	0.20682 (17)	0.63060 (4)	0.31478 (15)	0.0212 (2)	
C4B	0.1720 (2)	0.67590 (4)	0.20732 (17)	0.0283 (3)	
H4BA	0.1655	0.7038	0.2782	0.042*	
H4BB	0.0472	0.6730	0.1098	0.042*	
H4BC	0.2806	0.6804	0.1640	0.042*	
C5B	0.20560 (19)	0.58659 (4)	0.23242 (15)	0.0236 (2)	
H5BA	0.1819	0.5893	0.1125	0.028*	
C6B	0.23301 (18)	0.53901 (4)	0.29478 (16)	0.0236 (2)	

### Atomic displacement parameters (Å^2^)

**Table:**

	*U*^11^	*U*^22^	*U*^33^	*U*^12^	*U*^13^	*U*^23^
O1A	0.0363 (5)	0.0169 (4)	0.0265 (4)	0.0020 (3)	0.0138 (4)	0.0052 (3)
O21A	0.0920 (11)	0.0391 (6)	0.0227 (5)	−0.0056 (6)	−0.0006 (6)	0.0072 (4)
O22A	0.1363 (15)	0.0502 (7)	0.0365 (6)	0.0110 (8)	0.0483 (8)	−0.0054 (5)
O41A	0.0938 (10)	0.0162 (4)	0.0481 (6)	−0.0019 (5)	0.0378 (7)	−0.0017 (4)
O42A	0.0815 (9)	0.0244 (5)	0.0330 (5)	−0.0010 (5)	0.0272 (6)	0.0081 (4)
O61A	0.0723 (8)	0.0370 (6)	0.0193 (5)	0.0035 (5)	0.0023 (5)	−0.0045 (4)
O62A	0.0584 (7)	0.0192 (4)	0.0337 (5)	0.0086 (4)	0.0150 (5)	−0.0019 (4)
N2A	0.0604 (8)	0.0305 (6)	0.0191 (5)	−0.0096 (5)	0.0139 (5)	−0.0011 (4)
N4A	0.0437 (6)	0.0171 (4)	0.0323 (5)	0.0016 (4)	0.0190 (5)	0.0044 (4)
N6A	0.0378 (6)	0.0195 (4)	0.0210 (5)	−0.0017 (4)	0.0119 (4)	−0.0019 (3)
C1A	0.0215 (5)	0.0170 (4)	0.0183 (5)	0.0017 (4)	0.0075 (4)	0.0011 (3)
C2A	0.0325 (6)	0.0205 (5)	0.0165 (5)	−0.0013 (4)	0.0093 (4)	0.0003 (4)
C3A	0.0355 (6)	0.0173 (5)	0.0231 (5)	−0.0019 (4)	0.0127 (5)	−0.0020 (4)
C4A	0.0315 (6)	0.0143 (4)	0.0246 (5)	0.0011 (4)	0.0137 (5)	0.0031 (4)
C5A	0.0277 (5)	0.0182 (4)	0.0197 (5)	0.0015 (4)	0.0105 (4)	0.0024 (4)
C6A	0.0251 (5)	0.0153 (4)	0.0184 (5)	0.0008 (4)	0.0083 (4)	−0.0009 (3)
C7B	0.0506 (9)	0.0219 (5)	0.0345 (7)	0.0020 (5)	0.0146 (7)	−0.0077 (5)
N1B	0.0317 (5)	0.0142 (4)	0.0306 (5)	0.0025 (4)	0.0120 (4)	0.0028 (3)
N2B	0.0366 (6)	0.0156 (4)	0.0234 (5)	0.0027 (4)	0.0115 (4)	−0.0005 (3)
C1B	0.0352 (7)	0.0209 (5)	0.0322 (6)	0.0049 (5)	0.0185 (5)	0.0059 (4)
C2B	0.0371 (7)	0.0217 (5)	0.0213 (5)	0.0045 (5)	0.0104 (5)	0.0008 (4)
C3B	0.0232 (5)	0.0169 (4)	0.0232 (5)	0.0010 (4)	0.0078 (4)	0.0019 (4)
C4B	0.0371 (7)	0.0194 (5)	0.0297 (6)	0.0039 (5)	0.0135 (5)	0.0070 (4)
C5B	0.0308 (6)	0.0190 (5)	0.0198 (5)	0.0013 (4)	0.0074 (5)	−0.0004 (4)
C6B	0.0252 (5)	0.0170 (4)	0.0269 (5)	−0.0002 (4)	0.0071 (5)	−0.0027 (4)

### Geometric parameters (Å, °)

**Table:**

O1A—C1A	1.2438 (13)	C7B—H7BB	0.9800
O21A—N2A	1.2165 (17)	C7B—H7BC	0.9800
O22A—N2A	1.2219 (18)	N1B—C6B	1.3202 (16)
O41A—N4A	1.2281 (15)	N1B—C1B	1.4524 (16)
O42A—N4A	1.2338 (15)	N1B—H1BC	0.8800
O61A—N6A	1.2223 (15)	N2B—C3B	1.3235 (15)
O62A—N6A	1.2257 (14)	N2B—C2B	1.4512 (15)
N2A—C2A	1.4597 (15)	N2B—H2BC	0.8800
N4A—C4A	1.4332 (14)	C1B—C2B	1.5199 (18)
N6A—C6A	1.4558 (14)	C1B—H1BA	0.9900
C1A—C2A	1.4496 (15)	C1B—H1BB	0.9900
C1A—C6A	1.4504 (15)	C2B—H2BA	0.9900
C2A—C3A	1.3692 (16)	C2B—H2BB	0.9900
C3A—C4A	1.3918 (16)	C3B—C5B	1.3935 (15)
C3A—H3AA	0.9500	C3B—C4B	1.5055 (16)
C4A—C5A	1.3929 (15)	C4B—H4BA	0.9800
C5A—C6A	1.3744 (15)	C4B—H4BB	0.9800
C5A—H5AA	0.9500	C4B—H4BC	0.9800
C7B—C6B	1.5031 (17)	C5B—C6B	1.4014 (16)
C7B—H7BA	0.9800	C5B—H5BA	0.9500
			
O21A—N2A—O22A	123.42 (13)	C6B—N1B—C1B	124.82 (10)
O21A—N2A—C2A	118.62 (12)	C6B—N1B—H1BC	117.6
O22A—N2A—C2A	117.95 (12)	C1B—N1B—H1BC	117.6
O41A—N4A—O42A	122.28 (11)	C3B—N2B—C2B	126.45 (10)
O41A—N4A—C4A	119.43 (11)	C3B—N2B—H2BC	116.8
O42A—N4A—C4A	118.29 (10)	C2B—N2B—H2BC	116.8
O61A—N6A—O62A	123.71 (11)	N1B—C1B—C2B	113.60 (10)
O61A—N6A—C6A	118.17 (10)	N1B—C1B—H1BA	108.8
O62A—N6A—C6A	118.12 (10)	C2B—C1B—H1BA	108.8
O1A—C1A—C2A	123.64 (10)	N1B—C1B—H1BB	108.8
O1A—C1A—C6A	124.58 (10)	C2B—C1B—H1BB	108.8
C2A—C1A—C6A	111.68 (9)	H1BA—C1B—H1BB	107.7
C3A—C2A—C1A	124.72 (10)	N2B—C2B—C1B	113.33 (11)
C3A—C2A—N2A	116.88 (10)	N2B—C2B—H2BA	108.9
C1A—C2A—N2A	118.39 (10)	C1B—C2B—H2BA	108.9
C2A—C3A—C4A	118.80 (10)	N2B—C2B—H2BB	108.9
C2A—C3A—H3AA	120.6	C1B—C2B—H2BB	108.9
C4A—C3A—H3AA	120.6	H2BA—C2B—H2BB	107.7
C3A—C4A—C5A	121.43 (10)	N2B—C3B—C5B	126.98 (10)
C3A—C4A—N4A	119.15 (10)	N2B—C3B—C4B	115.16 (10)
C5A—C4A—N4A	119.41 (10)	C5B—C3B—C4B	117.78 (10)
C6A—C5A—C4A	118.55 (10)	C3B—C4B—H4BA	109.5
C6A—C5A—H5AA	120.7	C3B—C4B—H4BB	109.5
C4A—C5A—H5AA	120.7	H4BA—C4B—H4BB	109.5
C5A—C6A—C1A	124.70 (10)	C3B—C4B—H4BC	109.5
C5A—C6A—N6A	117.03 (10)	H4BA—C4B—H4BC	109.5
C1A—C6A—N6A	118.26 (9)	H4BB—C4B—H4BC	109.5
C6B—C7B—H7BA	109.5	C3B—C5B—C6B	131.55 (11)
C6B—C7B—H7BB	109.5	C3B—C5B—H5BA	114.2
H7BA—C7B—H7BB	109.5	C6B—C5B—H5BA	114.2
C6B—C7B—H7BC	109.5	N1B—C6B—C5B	125.93 (11)
H7BA—C7B—H7BC	109.5	N1B—C6B—C7B	115.97 (11)
H7BB—C7B—H7BC	109.5	C5B—C6B—C7B	118.10 (11)
			
O1A—C1A—C2A—C3A	172.43 (12)	O1A—C1A—C6A—C5A	−173.26 (12)
C6A—C1A—C2A—C3A	−4.09 (18)	C2A—C1A—C6A—C5A	3.22 (17)
O1A—C1A—C2A—N2A	−6.66 (18)	O1A—C1A—C6A—N6A	5.39 (18)
C6A—C1A—C2A—N2A	176.82 (11)	C2A—C1A—C6A—N6A	−178.13 (10)
O21A—N2A—C2A—C3A	143.77 (15)	O61A—N6A—C6A—C5A	34.74 (17)
O22A—N2A—C2A—C3A	−35.4 (2)	O62A—N6A—C6A—C5A	−145.02 (12)
O21A—N2A—C2A—C1A	−37.07 (19)	O61A—N6A—C6A—C1A	−144.01 (13)
O22A—N2A—C2A—C1A	143.81 (15)	O62A—N6A—C6A—C1A	36.22 (16)
C1A—C2A—C3A—C4A	3.4 (2)	C6B—N1B—C1B—C2B	−55.05 (17)
N2A—C2A—C3A—C4A	−177.54 (12)	C3B—N2B—C2B—C1B	−45.65 (17)
C2A—C3A—C4A—C5A	−1.33 (19)	N1B—C1B—C2B—N2B	75.70 (14)
C2A—C3A—C4A—N4A	177.46 (12)	C2B—N2B—C3B—C5B	3.7 (2)
O41A—N4A—C4A—C3A	4.2 (2)	C2B—N2B—C3B—C4B	−172.89 (12)
O42A—N4A—C4A—C3A	−176.34 (13)	N2B—C3B—C5B—C6B	3.3 (2)
O41A—N4A—C4A—C5A	−176.96 (13)	C4B—C3B—C5B—C6B	179.87 (13)
O42A—N4A—C4A—C5A	2.48 (19)	C1B—N1B—C6B—C5B	6.9 (2)
C3A—C4A—C5A—C6A	0.53 (19)	C1B—N1B—C6B—C7B	−173.08 (13)
N4A—C4A—C5A—C6A	−178.27 (11)	C3B—C5B—C6B—N1B	13.0 (2)
C4A—C5A—C6A—C1A	−1.66 (19)	C3B—C5B—C6B—C7B	−167.02 (14)
C4A—C5A—C6A—N6A	179.67 (11)		

### Hydrogen-bond geometry (Å, °)

**Table:**

*D*—H···*A*	*D*—H	H···*A*	*D*···*A*	*D*—H···*A*
N1B—H1BC···O1A	0.88	1.98	2.8434 (13)	169
N2B—H2BC···O42A^i^	0.88	2.09	2.9657 (14)	176
C5A—H5AA···O22A^ii^	0.95	2.54	3.4570 (16)	162
C7B—H7BA···O1A	0.98	2.55	3.2817 (17)	131
C1B—H1BA···O62A	0.99	2.45	3.2861 (15)	142
C1B—H1BB···O1A^iii^	0.99	2.51	3.4477 (16)	158
C2B—H2BA···O61A^iv^	0.99	2.48	3.0678 (16)	117
C2B—H2BB···O1A^v^	0.99	2.46	3.3135 (16)	144
C4B—H4BA···O42A^i^	0.98	2.59	3.4692 (17)	149

Symmetry codes: (i) −*x*+1/2, *y*+1/2, −*z*+3/2; (ii) *x*, *y*, *z*+1; (iii) −*x*, −*y*+1, −*z*+1; (iv) −*x*+1, −*y*+1, −*z*+2; (v) −*x*+1, −*y*+1, −*z*+1.
